# Hydrocortisone concentration influences time to clinically significant healing of acute inflammation of the ocular surface and adnexa – results from a double-blind randomized controlled trial

**DOI:** 10.1186/1471-2415-14-64

**Published:** 2014-05-10

**Authors:** Nikolay Sergiyenko, Ludmila Sukhina, Pavel Bezdetko, Yuriy Kovalenko, Nikolai Nikitin, Matthias Merzbacher, Dorothea Groß, Ralf Kohnen

**Affiliations:** 1 Kiev City Ophthalmological Hospital, Kiev, Kharkov, Ukraine; 2 Donetsk City Clinical Hospital, Donetsk, Ukraine; 3 Department of Ophthalmology, Kharkov District Clinical Hospital, Kharkov, Ukraine; 4 C Girshman Clinical City Hospital, Kharkov, Ukraine; 5 RUSSLAN Clinical Research Ltd, North Humberside, UK; 6 RPS Research Germany GmbH, Nuremberg, Germany; 7 URSAPHARM Arzneimittel GmbH, Saarbrücken, Germany; 8 ReSearch Pharmaceutical Services, Inc, Fort Washington, PA, USA; 9 Psychology Department, University of Erlangen-Nuremberg, Nuremberg, Germany

**Keywords:** Ophthalmic corticosteroids, Hydrocortisone acetate, Ocular surface, Adnexa, Acute inflammation, Clinical study, Eye ointment, Adaptive design, Treatment outcome

## Abstract

**Background:**

The efficacy of topical ophthalmic corticosteroids depends upon small modifications in preparations, such as drug concentration.

The aim of this study was to confirm that hydrocortisone acetate (HC-ac) ophthalmic ointments of 2.5% and 1% are more effective than a 0.5% eye ointment.

**Methods:**

In this randomized, double-blind, placebo-controlled, parallel-group clinical study, the change of signs and symptoms of acute inflammation of the ocular surface and adnexa was evaluated in 411 subjects.

**Results:**

Median time to clinically relevant response as estimated by 50% reduction in clinical signs and symptoms (CSS) total score over the entire trial was similar for subjects treated with HC-ac 2.5% (73.5 h) and for subjects treated with HC-ac 1.0% (67.7 h) and was considerably and significantly longer for subjects treated with HC-ac 0.5% (111.8 h) [p < 0.001 for both dosages]. All trial medications were safe and well tolerated.

**Conclusion:**

Hydrocortisone acetate 2.5% and Hydrocortisone acetate 1% eye ointments are efficacious and safe treatments for acute inflammations of the ocular surface or adnexa, and showed significantly better efficacy than a control group treated with Hydrocortisone acetate 0.5% therapy.

**Trial registration:**

Current Controlled Trials ISRCTN15464650.

## Background

Allergic eye inflammation is a localized allergic condition that is frequently associated with rhinitis and occasionally with asthma but often observed as the only prevalent allergic sensitization. Ocular allergic symptoms are estimated to be present in 40%–80% of the affected individuals [1,2]. Allergic eye diseases include a collection of clinical entities with variable presentation [3]. The most characteristic symptom of allergic conjunctivitis is itching, which is caused by the release of histamine and other mediators from specifically activated mast cells after allergen exposure [2,4,5]. Other signs and symptoms include redness, chemosis, tearing, a burning sensation, photophobia, papillary hypertrophy of the tarsal conjunctiva, eyelid and/or conjunctival edema [2]. Symptoms of seasonal allergic conjunctivitis (SAC) are generally more severe in comparison to perennial allergic conjunctivitis (PAC) [2,6].

On the other hand, blepharitis is an inflammatory condition of the eyelid margins that might be accompanied by an inflammation of the conjunctiva (blepharoconjunctivitis). The main goal of the treatment of allergic eye diseases is to reduce the inflammation early and to prevent complications, which can blur vision or cause dry eyes [2,7]. The most effective anti-inflammatory treatments are corticosteroids that are used in treating acute and severe forms of allergic reactions. Additionally, patients not responsive to other therapies benefit from corticosteroids [6,8].

Treatment of blepharitis, on the contrary, relies on eyelid hygiene, gentle lid massage, and warm compresses. Recently, the authors demonstrated the effectiveness of the antiseptic bibrocathol for the treatment of blepharitis [9]. Topical antibiotics might be used in patients who do not respond to eyelid hygiene and, in severe cases, oral antibiotics and topical steroids may also be beneficial [8].

Corticosteroids are potent anti-inflammatory drugs used for the treatment of allergic conjunctivitis and other non-infectious inflammatory diseases of the anterior eye. Topical ophthalmic steroids inhibit the production of various inflammation-causing mediators, such as prostaglandins, which are released when the eye reacts to allergens. Despite being important and effective therapeutic candidates for the treatment of allergic conjunctivitis [6], their use is typically reserved for patients not responsive to other therapies or for use in acute and severe forms of allergy because of the potential side-effects (e.g., increased intraocular pressure (IOP) and risk of cataract formation) [8]. We report the results from a randomized, controlled clinical trial to investigate whether higher concentrations of hydrocortisone acetate in the two eye ointments (i.e. Hydrocortisone acetate 2.5% and Hydrocortisone acetate 1%) are more effective in improving objective signs and subjective symptoms of non-infectious disease of the eye and ocular adnexa than the lower-concentrated Hydrocortisone acetate 0.5% eye ointment (concentration – response relationship).

## Methods

This was a multicenter, randomized, double-blind, parallel-group, active-control, study designed to evaluate the effects of HC-ac 2.5%, HC-ac 1.0%, and HC-ac 0.5% on relief of clinical signs and symptoms in patients with acute inflammation of the ocular surface or adnexa. The objective of the study was to show superior efficacy of at least one of the HC-ac dosages compared to the active control group, HC-ac 0.5%.

The eye ointments with Hydrocortisone acetate 2.5% and 1% are products of URSAPHARM Arzneimittel GmbH, Saarbruecken, Germany with the tradename Hydrocortison-POS® N 2.5% resp. 1%. The comparator product, eye ointment with 0.5% hydrocortisone acetate, is marketed as Ficortril® eye ointment 0.5% and manufactured by Dr. Mann Pharma, Berlin.

The study was conducted at 10 trial centers in the Ukraine according to the International Conference on Harmonisation - Good Clinical Practices guidelines (ICH-GCP) and in compliance with national Ukrainian law and regulations. This includes that each patient confirmed his/her informed consent in participating in this study in writing. The study was approved both by the Central Ethics Committee at Kyiv (Ukraine) and by the local ethics committees of each investigator.

Subjects were randomly assigned to one of the three active treatments in a ratio of 1:1:1. Research Pharmaceutical Services prepared a randomization list with balanced blocks of 6 subjects each, different blocks were used for subjects with or without pretreatment yielding a respective balance (stratification). Trial medication in all three treatment arms was of identical appearance, shape, and size; and the 3 medications were indistinguishable regarding consistency, color, smell, and viscosity.

Inclusion criteria were: Female or male outpatients; 18 to 75 years; presence of non-infectious disease of the eye and ocular adnexa, i.e. seasonal or perennial allergic conjunctivitis; acute allergic blepharitis; blepharoconjunctivitis; allergic lid edema; acute inflammation of the ocular surface, or adnexa for which topical steroid treatment was advisable. In addition, a minimum symptom intensity was required, as assessed by the “Clinical Signs and Symptoms” (CSS) total score ≥ 10 (maximum 30), and with at least one item scored of “moderate” or “severe” intensity.

Exclusion criteria were: Any systemic disease that prohibited steroid treatment; any contraindication for the use of steroids; pretreatment with systemic or topical steroids 1 month prior to, or concomitantly during trial participation. Further exclusion criteria were: Concomitant treatment with corticosteroids other than trial medication (e. g. corticosteroids for inhalation), mast-cell stabilizers, and other anti-allergens (except antihistamines, analgesics, anti-inflammatory drugs, anti-rheumatic drugs, and immunosuppressants); findings on fluorescein corneal staining at baseline (which prohibited steroid treatment); eye discharge (yellowish) with score ≥ 1 at baseline as assessed by the investigator; ocular injury and/or ocular surgery within 3 months prior to trial participation; wore contact lenses, and had a change in eye hygiene measures after study initiation.

The planned treatment duration was 14 days with 6 visits (Baseline [V0], control visits on Days 2 [V1], 4 [V2], 7 [V3], and end of trial visit if CSS = 0 on Day 10 ± 1 [V4], otherwise end of trial visit on Day 14 [V5]). 1 cm of eye ointment as randomized was administered in the lower conjunctival sac of each affected eye at baseline. The medication was applied twice daily (morning and evening at bedtime) for 10 consecutive days, with the option to prolong treatment to 14 days, until resolution of all signs and symptoms, or study end.

The primary objective of this trial was to demonstrate superiority of HC-ac 2.5% and 1.0% versus HC-ac 0.5% and, if possible superiority of HC-ac 2.5% versus HC-ac 1.0% regarding the time to 50% reduction in CSS total score. Secondary objectives were to assess further measures of efficacy, tolerability, and safety.

The CSS total score comprised 6 objective signs (conjunctival hyperemia, discharge, watering eyes, chemosis, red eyelids, swollen eyelids) and 4 subjective symptoms (foreign body sensation, itching, ocular pain, and dry-eye sensation) assessed at each trial visit (0–3 points per signs/symptoms, the higher the worse, maximum 30 points) (see Table 1). Objective signs were assessed by the investigator on the basis of a slit lamp examination of the eye with the highest CSS total score at baseline (if the score was equal for both eyes at baseline, one of the eyes was chosen deliberately) and subjective symptoms were assessed by interviewing the subjects.

**Table 1:** Grading of parameters to evaluate efficacy

**Signs and symptoms**	**Degree (=item value)**			
	**0**	**1**	**2**	**3**
**Symptoms**				
Foreign body sensation in the eye	not present	not disturbing	disturbing	painful
Itching	not present	not disturbing	disturbing	painful
Ocular pain	not present	not disturbing	disturbing	painful
Dry-eye sensation	absent	not disturbing	disturbing	intolerable
**Signs**				
Conjunctival hyperaemia	vessel normal	some vessel definitely injected	vessels injected more than 50%	all vessels injected
Discharge (yellowish)	not present	poorly visible	clearly visible	over 2 mm thick
Watering eyes	absent	Nnot disturbing	disturbing	Iintolerable
Chemosis	not present	poorly visible	clearly visible	very strong
Red eyelids	not present	poorly visible	clearly visible	very strong
Swollen eyelids	not present	poorly visible (not disturbing)	clearly visible (disturbing)	very strong (eye opening difficult)

As secondary efficacy variables the difference to baseline in CSS total score, the CSS subscores signs and symptoms, the time to first occurrence of at least 50% reduction in each CSS subscore, remitter (CSS = 0) rates at study end, the scores of individual CSS items at each trial visit, and the treatment duration until complete remission (CSS = 0) were analysed.

Safety parameters consisted of visual acuity test, IOP measurement (measured by tonometry) at visits V0, V4 and V5; adverse events (AEs); and global rating of tolerability (V4 and V5).

The data from subjects randomized and exposed were analysed (intent to treat [ITT] population). The time to first occurrence of at least 50% reduction in CSS total score was used to hierarchically test three hypotheses (H_1_: HC-ac 2.5% ≥ HC-ac 0.5%, H_2_: HC-ac 1% ≥ HC-ac 0.5%, and H_3_: HC-ac 2.5% ≥ HC-ac 1%) using Kaplan-Meier statistics and comparing each of the treatment groups pair-wise with the Cox-variant of the Log-Rank test. If ≥ 50% reduction was not reached during the time under treatment exposure, the subject was censored at the time of the last observation. The study followed a 2-stage adaptive design [10,11] with sample-size adjustment after the planned interim analysis. For the final hypotheses testing, the p-values of the 2 parts of the study were calculated separately and then combined using Fisher’s combination rule (p* = p1 × p2) and adjusted for type I error probability c_α_ ≤ 0.0038.

Secondary efficacy variables were compared between groups using ANCOVA with treatment as a factor and the baseline values as a covariate. The 2-sided 95% confidence intervals as well as the p-value for the baseline adjusted LS-means of all pair-wise differences between groups were calculated. Time to event analyses (50% improvement, CSS = 0) used the Kaplan-Meier-statistics and compared each of the treatment groups pair-wise with the Log-Rank-Test. Pair-wise comparisons of responders and remitters were performed with the chi^2^ test or Fisher’s exact test. Global ratings of efficacy by investigators and subjects were compared pair-wise using Wilcoxon U-tests.

Missing secondary efficacy variables were replaced using the last-observation-carried-forward (LOCF) approach.

The sample size estimation was based on the following assumptions: 10 or 14 days of observation; reduction of CSS total score by at least 50% in 85% of the subjects treated with HC-ac 2.5% and in 65% of subjects treated with HC-ac 0.5% after two days of treatment; significance level α = 0.025 (one-sided); power = 90%; drop-out rate during 10 days = 5%. This yielded, under the assumption of an exponential model, 3 × 67 subjects to be included in the comparative analysis. According to the interim analysis within the 2-stage adaptive design that was performed by an independent statistician, the trial could not be terminated prematurely due to rejection of the null hypothesis or futility. A recalculation of the sample size requested at least further 50 subjects per arm to be included into a second part of the study. To be on the safe side, it was decided to include 70 additional subjects per arm in the second part of the study, leading to a total of 411 subjects in the trial.

## Results

A total of 411 subjects were enrolled in the study and randomized to the HC-ac 0.5% (n = 133), the HC-ac 1% (n = 140), or the HC-ac 2.5% (n = 138) arms. All of the subjects received at least one dose of the trial medication and were available for the safety (SAF) and efficacy (intent-to-treat [ITT]) analysis (SAF and ITT populations, respectively).

A total of 398 subjects completed the study: 129 in the HC-ac 0.5% arm, 135 in the HC-ac 1% arm, and 134 in the HC-ac 2.5% arm. The remaining 13 subjects prematurely discontinued treatment during the first part of the study: 4 in the HC-ac 0.5% arm, 5 in the HC-ac 1% arm, and 4 in the HC-ac 2.5% arm. Main reasons for discontinuation were request of the subjects (n = 11; 4 in the HC-ac 0.5% arm, 4 in the HC-ac 1% arm, and 3 in the HC-ac 2.5% arm) or AEs (n = 2; 1 in the HC-ac 1% arm, and 1 in the HC-ac 2.5% arm).

Baseline demographic and clinical characteristics are summarized in Table 2.

**Table 2:** Demographic data at baseline (ITT)

**Demographic variables**	**Statistic**	**HC-ac 0.5% n = 133**	**HC-ac 1.0% n = 140**	**HC-ac 2.5% n = 138**
Sex Male	n (%)	58 (43.6)	61 (43.6)	56 (40.6)
Female	n (%)	75 (56.4)	79 (56.4)	82 (59.4.)
Age (years)	Mean ± SD	46.5 ± 17.2	49.36 ± 15.5	47.0 ± 16.3
	Median	47	50	48
	Range	17 – 76	18 – 75	20 – 75
BMI (kg/m^2^)	Mean ± SD	25.78 ± 3.93	25.76 ± 3.26	25.60 ± 3.99
	Median	25.54	25.86	25.40
	Range	17.30-40.80	19.10-35.02	17.01-44.78
Study eye Left	n (%)	59 (44.4)	62 (44.3)	68 (49.3)
Right	n (%)	74 (55.6)	78 (55.7)	70 (50.7)
Diagnosis				
Allergic conjunctivitis	n (%)	22 (16.5)	25 (17.9)	28 (20.3)
Allergic blepharitis	n (%)	45 (33.8)	59 (42.1)	56 (40.6)
Allergic blepharo-conjunctivitis	n (%)	66 (49.6)	56 (40.0)	54 (39.1)

BMI = body mass index; n = number of subjects; % percent of subjects in each group; SD: standard deviation.

Median treatment duration was 10 days in the 2.5% and 1.0% HC-ac and 11 days in the HC-ac 0.5% arm.

### Efficacy

**Figure 1:**
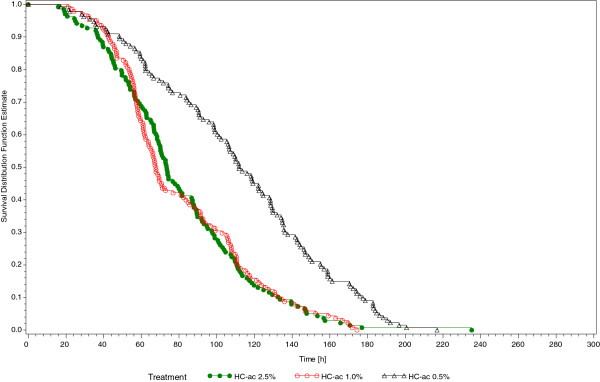
Time to ≥ 50% reduction in CSS total score.

**Figure 2:**
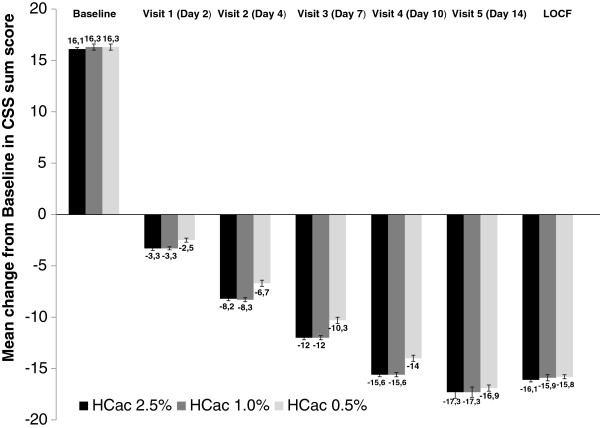
Change from baseline to all post-baseline visits in CSS total score.

The results of the three hypotheses related to the primary efficacy variable demonstrated that median time as estimated by the Kaplan-Meier statistic to 50% reduction in CSS total score over the entire trial (ITT, N = 411) was similar for subjects treated with HC-ac 2.5% (73.5 h) and for subjects treated with HC-ac 1.0% (67.7 h) and was considerably and significantly longer for subjects treated with HC-ac 0.5% (111.8 h) [p < 0.001] (Figure 1).Baseline mean values of CSS sum score were very similar between treatments (HC-ac 2.5%: 16.1 points, HC-ac 1.0%: 16.3 points, and HC-ac 0.5%: 16.3 points). The mean reduction of the CSS total score between each trial visit and baseline increased progressively for all treatments (Figure 2); however, this reduction was higher in subjects treated with HC-ac 2.5% and HC-ac 1% compared with subjects treated with HC-ac 0.5% from Day 2 (Visit 1) to Day 10 (Visit 4) [p ≤ 0.001]. In addition, subjects treated with HC-ac 2.5% experienced a greater reduction in the score than subjects treated with HC-ac 0.5% in the last observation carried forward (LOCF) analysis (p = 0.022).

The time (h) to first occurrence of at least 50% reduction in CSS total sum score and the two subscores (Table 3, Figure 1), as well as the time to complete remission (Table 3) was shorter when being treated with HC-ac 2.5% and 1% than when being treated with HC-ac 0.5% (p < 0.001). There was no difference in these time-to-event analyses between the two concentrations of HC-ac 1.0% and 2.5%.

**Table 3:** Time (hours) to ≥50% reduction and to complete remission in CSS sum and subscores

**Statistic**	**HC-ac 0.5% n = 133**	**HC-ac 1.0% n = 140**	**HC-ac 2.5% n = 138**
**Time to 50% reduction from baseline (days)**			
CSS total			
Mean ± SD	112 ± 47	81 ± 36	81 ± 38
Median	112	68	74
Range	17 – 217	21 – 174	16 – 235
CSS objective signs			
Mean ± SD	114 ± 51	83 ± 41	83 ± 39
Median	121	72	82
Range	15 – 272	18 – 238	16 – 183
CSS subjective symptoms			
Mean ± SD	110 ± 48	82 ± 41	80 ± 42
Median	110	68	69
Range	17 – 224	18 – 253	14 – 266
**Time to complete remission (days)**			
CSS Total = 0			
Mean ± SD	250 ± 55	232 ± 45	228 ± 39
Median	240	220	220
Range	70 – 342	72 – 379	121 – 342
CSS objective signs subscore = 0			
Mean ± SD	249 ± 56	232 ± 45	227 ± 40
Median	240	220	220
Range	70 – 342	72 – 379	121 – 342
CSS subjective symptoms subscore = 0			
Mean ± SD	248 ± 586	224 ± 52	220 ± 48
Median	240	220	219
Range	70 – 342	72 – 379	70 – 342

*CSS* Clinical signs and symptoms n = number of subjects; *SD* standard deviation; range: minimum – maximum.

Almost all subjects had responded to treatment medication (CSS improvement by ≥ 50%) on Day 10 (Table 4) and no notable differences were observed among treatments. No differences were observed between the proportions of remitters (CSS = 0) in the HC-ac 2.5% and HC-ac 1% treatment groups. However, the proportion of remitters was higher in both the HC-ac 2.5% and HC-ac 1.0% treatment groups than in the HC-ac 0.5% treatment group (p < 0.001).

**Table 4:** Responder and remitter rates after 10 days of treatment

**Visit**	**Statistic**	**HC-ac 0.5% n = 133**	**HC-ac 1.0% n = 140**	**HC-ac 2.5% n = 138**
Responders	n (%)	129 (97.0)	136 (97.1)	135 (97.8)
Remitters	n (%)	76 (57.1)	116 (82.9)	119 (86.2)

n = number of subjects; % percent of subjects in each group.

A responder was defined by a ≥ 50% improvement in the CSS total score between Day 10 visit and baseline.

A remitter was defined by a CSS total score = 0 (symptom-free) at the Day 10 visit.

At baseline, mean average scores for each of the subjective symptoms and objective signs decreased progressively in all treatment groups throughout the study visits achieving maximum improvement on Day 10. Again, this reduction was greater when being treated with HC-ac 2.5% and 1% than when being treated with HC-ac 0.5% for the following items: foreign body sensation (p < 0.001 both), itching (p < 0.001 both) and dry eye (p = 0.004 both), as well as conjunctival hyperemia (p < 0.001 both), chemosis (p = 0.009 and p = 0.011, respectively), red eye lids (p < 0.001 both), and swollen eye lids (p < 0.001 both). The reduction of yellowish discharge was greater only in subjects treated with HC-ac 2.5% than those treated with HC-ac 0.5%.

The patients were treated in different seasons of the year which correlated with the severity of symptoms at baseline and the time of reduction of symptoms. However, an influence of the season on the differences of the three treatments could not be observed.

All trial medications were safe and well tolerated. No deaths or serious adverse events (SAEs) were reported throughout the study, and the incidence of subjects with AEs was 2.2%. In two subjects, the AEs lead to treatment interruption and discontinuation. One of these subjects was treated with HC-ac 2.5% and experienced increased lacrimation (mild); the other was treated with HC-ac 1% and had conjunctival hyperemia (moderate) and increased lacrimation (mild). Other AEs reported during the study were discomfort or pruritus at the site of application. No clinically relevant changes during treatment were observed in visual acuity measures and intraocular pressure (IOP) values.

## Discussion

This multicenter, randomized, double-blind, parallel-group, active-controlled study in subjects with acute inflammation of the ocular surface or adnexa could demonstrate a faster (by 31 hours) clinically relevant (50% of baseline severity) improvement of objectively determined signs and subjectively reported symptoms when treated with two hydrocortisone eye ointments in concentrations of 2.5 and 1.0% (HC-ac 2.5%, HC-ac 1.0%) compared with a lower concentrated preparation (HC-ac 0.5%). There was no difference in this primary endpoint between the 1.0% and the 2.5% concentration. This observation indicates that there is no linear dose–response-relationship for this type of treatment. All secondary outcome measures consistently supported the primary finding, showing improvement in the total CSS score, its subscores for objective signs and subjective symptoms, and in most of the individual signs and symptoms. Of note, the rate of symptom-free subjects after the maximum treatment duration of 10 days was 83% and 86% in the 1.0% and the 2.5% concentration arms, respectively: these rates were approximately 25% higher than those in the control group (57%). At the end of the 2-week treatment period, with a few exceptions (HC-ac 0.5%: 12.3%, HC-ac 1.0%: 6.4%; HC-ac 2.5%: 2.2%) all subjects were symptom-free.

Topical eye ointments are available with different concentrations of hydrocortisone acetate. The findings of this study indicate that treatment with corticosteroids is highly effective in subjects with allergic conjunctivitis [8]; however, they also show that higher concentrations of hydrocortisone accelerate early onset of clinically relevant improvement (2.8 [1%] and 3.1 [2.5%] days versus 4.7 [0.5%] days, median); time to complete remission (9.2 [1% and 2.5%] days versus 10.0 [0.5%] days) and in the rates of symptom-free subjects ten days after start of treatment (see above). In all analyses, treatment with 1% and 2.5% hydrocortisone helped to gain approximately one day with less severe or no symptoms compared to the eye ointment with the lowest concentration. The advantage of more than 1.5 days of the two Hydrocortisone acetate concentrations in the time to response is expressed in the hazard ratios that indicate a 2.4-fold (95%-CI: 1.7;3.3; HC-ac 2.5%) or a 2.2-fold (95%-CI: 1.6; 3.1; HC-ac 1.0%) higher chance to achieve an early relevant relief (≥50% of baseline severity) from symptoms than under the lower-concentrated hydrocortisone.

Regarding the relevance of allergic ocular diseases on quality of life or work productivity [12], such a faster recovery, could imply a significant reduction of indirect health costs as well as shorter duration of burdens of the disease for the affected subjects. Such data were not included in our study design; however, if in accordance with the clinical data, they would support the importance of the described differences between the two Hydrocortisone acetate treatments and the Hydrocortisone acetate 0.5% treatment even more strongly, and are suggested to be included in future trials.

The desired rapid treatment success could be achieved by doubling the concentration of 0.5% (Hydrocortisone acetate) to 1% (HC-ac 1.0%) hydrocortisone, a further increase of hydrocortisone concentration to 2.5% (HC-ac 2.5%) did not contribute additional substantial benefit.

**Figure 3:**
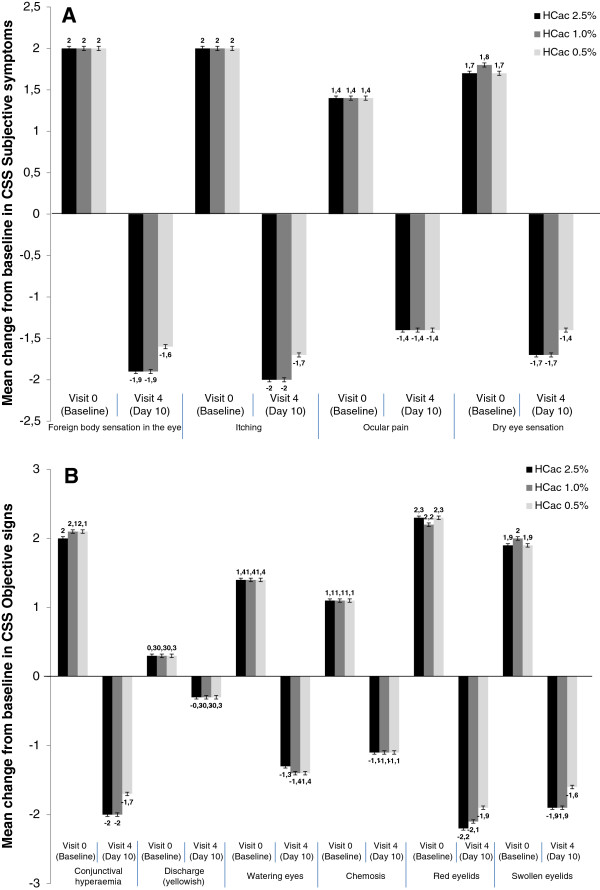
Change from baseline to Day 10 in CSS total score for subjective symptoms (A) and objective symptoms (B).

Tolerability was not a major treatment issue, with only 2.2% of subjects in total experiencing mild to moderate AEs: increased lacrimation, conjunctival hyperemia, and pruritus/discomfort at the application site. Only two subjects discontinued from the study prematurely because of AEs. In addition, visual acuity and IOP values were within the normal ranges at baseline and at the end of the study, and no clinically relevant changes were reported throughout the 2-week treatment duration. No instances of cataract formation were observed.Subjects with baseline moderate to severe symptoms of acute inflammation of mostly allergic blepharoconjunctivitis (43%), as well as allergic blepharitis (39%) or allergic conjuncitivitis (18%) were treated. Most severe symptoms were foreign body sensations in the eye, itching, red or swollen eyelids, and conjunctival hyperemia (Figure 3), whereas yellowish discharge was present in only a few subjects. Non-response to other therapies was not an inclusion criterion; therefore, our conclusions cannot be extended to this treatment-refractory sub-population of subjects with allergic conjunctivitis.

## Conclusion

We conclude from this trial that hydrocortisone eye ointment in higher concentration of at least 1% is superior to a frequently used 0.5% concentration with regard to earlier onset of clinically relevant improvement and shorter time to complete healing in subjects with acute non-infectious inflammation of the ocular surface or adnexa. The higher concentrations, 2.5% and 1% were equally effective. All hydrocortisone concentrations were safe and well tolerated, none of the known risks of ketone-corticosteroids were observed during this 2-week treatment period.

## Abbreviations

AE: Adverse event; CSS: Clinical signs and symptoms; HC: Hydrocortisone; HC-ac: Hydrocortisone acetate; IOP: Intraocular pressure; ITT: Intent- to- treat.

## Competing interests

This clinical trial was sponsored by URSAPHARM Arzneimittel GmbH, Germany. URSAPHARM Arzneimittel GmbH is the manufacturer of Hydrocortison-POS N 1% and Hydrocortison-POS N 2.5% eye ointments.

## Authors’ contributions

SN, LS, PB and YK participated in the design of the study, acquisition of data and interpretation of data. NN made substantial contribution to the acquisition of data. MM and RK made substantial contribution to the design of the study, performed the statistical analysis and interpretation of the data and drafted the manuscript. DG gave final approval to the design of the study and interpretation of data. All authors have given final approval of the version to be published.

## Pre-publication history

The pre-publication history for this paper can be accessed here:

http://www.biomedcentral.com/1471-2415/14/64/prepub
